# Human’s Intuitive Mental Models as a Source of Realistic Artificial Intelligence and Engineering

**DOI:** 10.3389/fpsyg.2022.873289

**Published:** 2022-05-30

**Authors:** Jyrki Suomala, Janne Kauttonen

**Affiliations:** ^1^NeuroLab, Laurea University of Applied Sciences, Vantaa, Finland; ^2^Competences, RDI and Digitalization, Haaga-Helia University of Applied Sciences, Helsinki, Finland

**Keywords:** computational meaningfulness, intuitive models, brain’s valuation network, artificial general intelligence, neuroeconomics

## Abstract

Despite the success of artificial intelligence (AI), we are still far away from AI that model the world as humans do. This study focuses for explaining human behavior from intuitive mental models’ perspectives. We describe how behavior arises in biological systems and how the better understanding of this biological system can lead to advances in the development of human-like AI. Human can build intuitive models from physical, social, and cultural situations. In addition, we follow Bayesian inference to combine intuitive models and new information to make decisions. We should build similar intuitive models and Bayesian algorithms for the new AI. We suggest that the probability calculation in Bayesian sense is sensitive to semantic properties of the objects’ combination formed by observation and prior experience. We call this brain process as computational meaningfulness and it is closer to the Bayesian ideal, when the occurrence of probabilities of these objects are believable. How does the human brain form models of the world and apply these models in its behavior? We outline the answers from three perspectives. First, intuitive models support an individual to use information meaningful ways in a current context. Second, neuroeconomics proposes that the valuation network in the brain has essential role in human decision making. It combines psychological, economical, and neuroscientific approaches to reveal the biological mechanisms by which decisions are made. Then, the brain is an over-parameterized modeling organ and produces optimal behavior in a complex word. Finally, a progress in data analysis techniques in AI has allowed us to decipher how the human brain valuates different options in complex situations. By combining big datasets with machine learning models, it is possible to gain insight from complex neural data beyond what was possible before. We describe these solutions by reviewing the current research from this perspective. In this study, we outline the basic aspects for human-like AI and we discuss on how science can benefit from AI. The better we understand human’s brain mechanisms, the better we can apply this understanding for building new AI. Both development of AI and understanding of human behavior go hand in hand.

## Introduction

The development of artificial intelligence (AI) and its application to the engineering has been tremendous in the 2000s, and particularly during the past 10 years. Much of this progress has come from advances in “deep learning,” which refers to multilayer network-style models that emulate the working principles of the brain. Today, AI can outperform humans on certain narrow tasks previously thought to require human expertise, such as playing chess, poker and Go (Schrittwieser et al., 2020), object recognition (LeCun et al., 2015), natural language understanding (He et al., 2021), and speech recognition (López et al., 2017; Bengio et al., 2021). In addition, self-driving cars, goods transport robots, and unmanned aircrafts will soon be a part of normal traffic (Hancock et al., 2019). Despite these successes, we are still far away from artificial general intelligence (AGI), which is a broader type of AI that can learn to perform at or above the human level across a wide variety of tasks (Legg and Hutter, 2007). This is in comparison to currently available narrow AI models that can do specific tasks better than humans, but cannot generalize to many different tasks. There are various types of AGI, but in this study, we ficus on AGIs comparable to human-like intelligence. The current computers still struggle to emulate the high flexibility of human mind. The human mind has evolved to excel at flexibility solving many different problems approximately rather than solving a small number of specific problems precisely (Gershman, 2021a). On the contrary, today’s AI can solve specific problems accurately and quicker than humans.

Moreover, human can learn based on a few examples, whereas AI needs huge amount of learning trials to reach comparable performance (Bengio et al., 2021). This study focuses in explaining human behavior from intuitive mental models’ perspectives. We describe essential features of human reasoning from the current computational resource rational model of mind. This approach proposes that the humans are intuitive statisticians and meaningfulness calculators, who use Bayesian computation to model contexts under uncertainty (Gershman et al., 2015; Friston et al., 2021). Bayesian theory provides a principled way for calculating a conditional probability. In its core is the Bayes’ theorem that states that the conditional probability of an event, based on the occurrence of another event, is equal to the likelihood of the second event given the first event multiplied by the probability of the first event (Barber, 2012). In the context of human decision making, the Bayesian theory posits that the brain is a prediction machine that is automatically matching incoming sensory data with an inner model of the world based on prior experience (Cohen et al., 2017; Friston et al., 2021). Bayesian decision theory involves many different approaches; however, in this study, we focus on human intuitive reasoning from Bayesian theory perspective, when an individual tries to find optimal solutions by using computational meaningfulness. By computation, we mean the process by which the brain changes its own mental model of the world (Tegmark, 2017) based on information from the environment to behave in optimal ways. We hypothesize that this is accomplished by Bayes’ rules with some approximation inference, such as sampling. We call this brain process as computational meaningfulness because Bayesian computation as the current, ongoing context is included into the estimation of the probability distribution of the task object.

In this study, we discuss research related to artificial intelligence, behavioral sciences and neurosciences, and how these fields can help us in pursuing human-like AGI. Our selection of literature includes works which combine human’s tendency to interpret behaviorally relevant aspects of the environment based on the intuitive mental models; computation based on Bayesian theory and AI. In addition, we have chosen literature that represent neuroscience studies that demonstrate the properties of the human brain’s valuation and default network as essential computational adaptive biological device. The selected literature includes seminal and topical works related to all the key concepts that make the computational meaningfulness framework and its components. In Table 1, we have listed the key concepts that we consider essential toward pursuing human-like AGI and are discussed in this this study. Our rationale of the concepts is as follows. First, to make sense of the environment, a human (or an artificial agent) needs mental models of the world, which include intuitive physics, psychology, and culture. These are the priors that are needed for probabilistic predictions for events and actions. Second, the mental models can be learned by observing and acting on the world, which can be achieved *via* reinforcement and self-supervised learning techniques. Having the mental models, optimal decisions are made *via* Bayesian inference and meaningful reasoning to estimate posterior probabilities. Mental models are updated as needed to improve predictions (i.e., reduce prediction errors) for future actions. At the core of this framework is the Bayesian reasoning, which we consider as the most promising and plausible mechanism for contextual decision making and reasoning. Third, in human brains, many of the above complex computations are performed in a “hardware” which in neuroscience are known as default mode and valuation networks. We consider brain as an over-parametrized modeling organ that performs Bayesian computations *via* approximate sampling principle.

**TABLE 1:** Key concepts from artificial intelligence, behavioral sciences and neurosciences from Bayesian theory perspective as discussed in this study.

Concept	Description	Impact and relationship to Bayesian theory	References
Intuitive physics (Section “Intuitive Physics”)	Physical, immutable constraints of the environment and world.	Mental models (priors) of the world. Predictions are based on combining prior beliefs with upcoming events.	Battaglia et al., 2013; Lake et al., 2017
Intuitive psychology (Section “Intuitive Psychology”)	Understanding of self and others in the local environment.		Lake et al., 2017; Sapolsky, 2018
Intuitive culture (Section “Intuitive Culture”)	Contextual constraints for behavior and principles of the environment on large scale.		Sapolsky, 2018
Reinforcement learning (Section “Reinforcement and Self-Supervised Learning”)	Mechanism to learn from actions and their rewards.	Creation, usage, and updating of the mental models. Computation of posterior probabilities of events using sensory information and priors.	Lake et al., 2017; Silver et al., 2021
Self-supervised learning (Section “Reinforcement and Self-Supervised Learning”)	Mechanism to learn by observing the world.		Sekar et al., 2020; Levine, 2022
Bayesian inference (Section “The Bayesian Brain and Meaningful Reasoning”)	Mechanism to combine incoming data with mental models (priors).		Gershman et al., 2015; Sanborn and Chater, 2016
Meaningful reasoning (Section “The Bayesian Brain and Meaningful Reasoning”)	Contextual decision-making strategies in varying situations.		Jaynes, 2003; Suomala, 2020
Default mode network (Section “Brains as Over-Parameterized Modeling Organ and the Role of Default-Mode Network”)	Integrates high-dimensional information and keeps track of ongoing events and contexts.	Neurophysiological mechanisms of decision making. Does over-parametrized, contextual computations with Bayesian sampling.	DuBrow et al., 2017; Yeshurun et al., 2021
Valuation network (Section “The Brain’s Valuation Network”)	Computes and predicts the relative value of items and decisions.		Genevsky et al., 2017; Magrabi et al., 2021

This study is organized as follows. We start from intuitive mental models (Section “Intuitive Mental Models”) that are at the core of the ability of AGI to generalize over different tasks. Then, we describe how such models are created and applied to make predictions in rich and complex environments (Sections “Intuitive Mental Models” and “Brain as a Prediction Machine”). We make connections with neuroscientific and neuroeconomics research. Sections “Intuitive Mental Models” and “Brain as a Prediction Machine” serve mostly as an overview of the literature and the theoretical concepts. The key concepts are listed in Table 1 with corresponding section numbers included. In Section “Comparing Brain Model With Current AI Models,” we look at shortcoming of current AI models and compare then with brain models. Finally, in “Discussion,” we discuss the major gaps in current AI models, our findings, sketch the path for future research, and the importance of computational meaningfulness. We end with a summary and conclusions in a Section of Summary and Conclusions.

## Intuitive Mental Models

The environments a person encounters contain an almost infinite amount of information. Therefore, the information that reaches the brain is often highly ambiguous. The current research emphasizes that the human brain resolves this uncertainty by using contextual information and prior experiences (Gershman, 2021a). Prior experiences are combination of intuitive mental models in the brain and learned new knowledge and skills relating to the world acquired through experience.

Prior experiences are causally structured representations coded in the brain. These representations are organized by the information of the world by according to the general principles that allows them to generalize across the varied scenarios in the new contexts (Spelke and Kinzler, 2007). Whereas the previous studies have emphasized that the representations are based on the pattern recognition, but the current research emphasizes that the representations are better understood in terms of the dynamic models with limited data (Lake et al., 2017). Thus, a human can use and learn to enrich mental models in flexible and suitable ways in varied contexts. A person does not learn the names and other concepts of objects in a vacuum but in relation to a context and other objects. Prior experiences are based on theory-like dynamic mental models in a person’s mind. These mental models help a person to constrain information flow and choose most essential information in each situation by inductive bias (Baum, 2004; Suomala, 2020; Gershman, 2021a).

Dynamic mental models include causal roles and functions of objects. Causality can also bind some features of objects together, explaining why some features such as “can fly,” “has wings,” and “has feathers” co-occur across objects, whereas others do not (Lake et al., 2017). Human-level scene understanding involves composing a story that explains the observations, drawing upon and integrating the ingredients of intuitive physics, intuitive psychology, and intuitive culture. Pure perception without these objects’ functions and causal and other relationships (like co-occurrence) between these objects, can lead to revealing errors. The understanding of objects’ functions in a specific context is lacking in today’s AI. When the image captions are generated by a deep neural network, it gets the key objects in a scene correct, but fails to understand the relationships between objects and people in the image, or the causal relationships between the objects. In other words, the deep neural network does not build the right dynamic model of the data (Lake et al., 2017).

Inductive bias is a person’s predisposition to use prior experiences to interpret a context in optimal way. Learning new things requires expectations about the essential things of each situation in relation to the irrelevant things. Therefore, the use of inductive biases is an indispensable property of rationality of human’s mind (Gershman, 2021a). Already, babies have developed clear mental models and inductive biases, which helps them to learn to behave in their environments. Babies have intuitive mental models about physics, psychology, and culture. A child begins to learn to like certain fairy tales as early as fetal age, as Anthony DeCasper has shown in his famous “The-Cat-in-the-Hat” study (DeCasper and Spence, 1986).

Next, we describe three essential intuitive models, which guide children’s behavior and learning at the early stage of the development. These intuitive models are related to physics, psychology, and culture. We use the term intuitive here to refer to a type of commonsense knowledge or “start-up software” that humans learn during their development toward adulthood and which allows us to learn new tasks and adapt information (Lake et al., 2017; Bengio et al., 2021). While constraints set by physics are fixed and same for all humans, cultural aspects are flexible and depend both on location (i.e., country) and time (i.e., era). Nevertheless, all factors can be considered intuitive in a sense that together they form mental models relevant for humans. To create mental models themselves, the following two key mechanisms have been suggested and applied successfully in the AI research: Reinforcement and self-supervised learning, which require observing and acting on environment. These are discussed in Subsection “Reinforcement and Self-Supervised Learning”.

### Intuitive Physics

Young children and even infants have a rich mental model of intuitive physics. These mental models allow them to know primitive object concepts. They understand on implicit level that an object will persist over time, that objects are solid and coherent. In addition, they can expect that inanimate objects to follow the principles of persistence, continuity, and cohesion (Spelke, 1990). Moreover, infants believe that the objects should move along smooth paths, not wink in and out of existence (Spelke, 1990). Equipped with these intuitive mental models about physics, children begin to make accurate predictions, learn more quickly, and behave in optimal ways. Although a task may be new for a child, physics still works the same way (Lake et al., 2017).

These predictions guide later learning and at around 6 months, infants have already developed different predictions for soft bodies, rigid bodies, and liquids. Whereas unity and organization for objects include a relation of unity and organizations governing their parts, concepts of substances do not include these properties (Rips and Hespos, 2015). At the early stage of the development, children have learned that while solid objects cannot to go through barriers, liquids can go (Rips and Hespos, 2015). During the first year, infants have gone through several transitions of comprehending basic physical concepts such as collisions, support, and containment (Lake et al., 2017).

According to the intuitive physics approach, people reconstruct a perceptual scene using internal model of the objects and their physically relevant properties. These properties are, for example, mass, surface friction, elasticity, gravity, friction, and collision impulses. The intuitive physical state model is approximate and probabilistic, and oversimplified and incomplete in many ways relative to physical ground truth (Lake et al., 2017). Still, it is rich enough to support peoples’ optimal behavior in their contexts and to support to embrace new more diverse and precise mental models. These intuitive physic models enable flexible adaptation to a wide range of everyday scenarios and judgments and support people to make hypothetical or counterfactual predictions in a way that goes beyond perceptual cues (Lake et al., 2017).

Intuitive physical model approach has proven correct in experiments, in which wooden blocks from the game Jenga has been used. In these experiments, the adult participants (Battaglia et al., 2013) and infants (Téglás et al., 2011) predict how a tower will fall. The findings show consistently that the infants can predict the movements of the wooden blocks according to similar ways than adults. Whereas an infant can learn and predict the movements of wooden blocks based on few examples, AI (PhysNet) requires extensive training—between 100,000 and 200,000 scenes—to answer the question will the tower fall or not? (Lake et al., 2017). Thus, a human requires far less repetitions as AI to perform any particular task and can generalize to many novel complex scenes with no new training required.

### Intuitive Psychology

A second intuitive mental models in the early development are related to intuitive psychology. By applying these models, infants understand that other people have mental states like beliefs and goals, and this understanding strongly constrains their learning and predictions.

Pre-verbal infants can distinguish animate agents from inanimate objects. This distinction is likely based on early–present detectors for sensory cues, such as the presence of eyes, motion initiated from rest, and biological motion (Schlottmann et al., 2006; Lake et al., 2017). Such cues often detect agency and infants also expect the agents to have goals, and to take efficient actions toward those goals. In addition, infants assume that the agents behave contingently and reciprocally (Spelke and Kinzler, 2007).

One essential part of an intuitive psychological model is the theory of mind, which helps children to understand the intentions of other people (Tomasello, 2014). Theory of mind helps a child to participate with others in collaborative activities with shared goals and intentions (Tomasello et al., 2005). These models can be socially directed, and these models allow a child to infer who are good and who are bad, or which virtual agent are good and bad. For example, infants begin to discriminate antisocial agents that hurt or hinder others from neutral agents at around 3 months of age (Lake et al., 2017).

Crucially, unlike in intuitive physics, model-based reasoning in intuitive psychology can be nested recursively to understand the social interactions and this way, we can reason about agents thinking about other agents (Lake et al., 2017). In this way, the intuitive psychology provides a child a basis for an efficient learning from others, especially in teaching settings with the goal of transforming culturally important knowledge efficiently. Thus, it is safe to argue that the infants expect agents to act in a goal-directed, efficient, and socially sensitive fashion (Spelke and Kinzler, 2007; Lake et al., 2017) by using intuitive psychological models.

Lake et al. (2017) are agnostic with regard to the origins of physical and psychological intuitive mental models. Whether they are innate, enriched, or rapidly learned, they are likely essential foundation to a later development and learning. However, current psychological (Tomasello et al., 2005; Tomasello, 2014) and neurobiological research (Geary, 2005; Sapolsky, 2018) emphasize that the most essential ingredient of the human mind is a unique motivation to share psychological and cultural states with others. These socially and culturally shared intentions are utterly intertwined with physical and psychological aspects of human mind (Sapolsky, 2018).

Thanks to evolution, humans are born with priors about ourselves, world and how to learn, which determine what categories of skills we can acquire and what categories of problems we can solve. These priors are the reason why humans can acquire certain categories of skills with remarkable efficiency. These priors range from low (e.g., sensorimotor), meta (spatio–temporal continuity) to high-level (e.g., theory of mind and 3D navigation) (Chollet, 2019). Therefore, we add intuitive culture as third ingredient as an essential part of human mental models. Next section describes it more specifically.

### Intuitive Culture

Whereas the early developed intuitive physics and psychology of infants are likely limited to reasoning about objects and agents in their immediate vicinity, the cultural values, artifacts, habits, and ideals develop later with interactions of child and other people and official institutions. However, the cultural dimensions of environment might be as essential in later ages of the development as the intuitive physics and psychology have at early ages. Unlike physics, which remains same for all humans and all times, the mental models related to culture depend strongly on where and when the development takes place.

The human brain as whole support an individual to organize and control his or her life in the ways that will enhance the expected standard of living in the culture in which each individual lives. This ability to “cast oneself as a player in scenarios emerging from various choices available at any given moment” (Geary, 2005, p. 200). These various choices are cultural possibilities, which are available for individuals. A unique aspect of this evolved ability is that human can formulate an autonoetic mental model of potential future states and to manipulate these models in ways that enable the simulation of control-related behavioral strategies (Geary, 2005). Thus goal-setting and meaningful interpretations of cultural contexts are essential aspects of human life, and these goals can help humans to prefer long-term options more than short-term ones. Thus, people choice by comparing the current situation to an autonoetic mental representation of a “perfect world” (Geary, 2005, p. 234). The perfect world is one in which the individual can organize and control his or her life in the ways that will enhance the expected standard of living.

We assume that the cultural situations might have similar effects than intuitive physics and psychology for human’s behavior. For example, thinking about meeting in office activate quickly and intuitively arrives at mental models about colleagues, tools, discussion topics, and other work-related issues (DuBrow et al., 2017). However, if you open the door of the office and see that there is birthday cake on the table, balloons in the air and you will hear happy music playing, you immediately and quickly infer and forms mental model of birthday party (DuBrow et al., 2017). Work and birthday contexts are cultural entities, which have strong effects for our behavior. In the similar way, when you think about the weekend, the summer holidays, and the next step in your career development, your brain can quickly and intuitively forms the mental models about these cultural contexts. This is possible because you have grown up in a particular culture where there is a meaningful interpretation of these contexts.

Lake et al. (2017) described the intuitive physics and psychology as essential core ingredients of human intelligence, learning and thought. However, they emphasize that these are hardly the only ingredients needed for human-like rationality. Their article is like a roadmap for human-like AGI and they assume that the intuitive physics and psychology is good starting point to build this kind of AI. What is missing from this approach, however, is the recognition that children’s developmental core ingredients of cognition are shaped by their culture-specific social and material environment. Children’s early and ontogenetically persistent experiences with their cultural environment affect what kind of intuitive mental models’ children develop (Clegg and Corriveau, 2017). Therefore, we added third ingredient, intuitive culture described above, which we assume is one essential source of human-like optimal behavior.

### Reinforcement and Self-Supervised Learning

Actions, either by oneself or others often results in feedback (rewards), which guide the learning. Learning *via* rewards is known as the reinforcement learning and it has been suggested recently that reward signal itself is enough for generating intelligence (Silver et al., 2021). With suitable rewards, the reinforcement learning paradigm could explain the intelligence on all levels, including perception, motor control, social interaction, language and—most importantly—generalization between different tasks (Silver et al., 2021). A successful agent needs to acquire behaviors that exhibit all these skills while learning to maximize the rewards in the world. Singular major goals, such survival (or success) in general in the world implicitly require the ability to achieve a wide variety of subgoals, and the maximization of one high-level reward should therefore be enough to yield an artificial general intelligence for wide range of rewards. The importance of the culture and social interaction in building world models cannot be underestimated as the effect of culture spans very long timespans in both human developments, but also in evolution (Friston et al., 2021). We argue that in this view, the culture where the agent lives, has central importance in defining what are the high-level rewards that drive development of intelligence. This contextual information necessarily affects and guides the decisions.

As the reinforcement learning is based on rewards, it often requires a very large number of interactions and iterations. This type of learning method tends to produce task-specific, specialized systems that are often brittle outside of the narrow domain they have been trained on (Bengio et al., 2021). For humans, the values associated with decisions are computed in special valuation network of the brain (see Subsection “The Brain’s Valuation Network”); hence, there is a connection with computations of reward signals in reinforcement learning (see Subsection “Intuitive Psychology”).

On the other hand, rewards are not always necessary and learning can also occur in self-supervised manner by simply observing the environment without explicit rewards. This framework is known as self-supervised learning, which has been highly successful in recent AI research and considered as a promising path toward powerful AI (Bengio et al., 2021; Levine, 2022). A self-supervised learning agent adopts supervisory signals that are inferred from the structure of the world (or data) itself. For example, by masking the individual words in sentences, small patches in images or short segments in speech, deep neural network models can learn representations of fundamental rules of the underlying data (Baevski et al., 2022). The recent successes of self-supervised learning include the development of advanced language models, where the neural network learns the meaning of words and the basic structure of the written language (He et al., 2021; Weidinger et al., 2021). Such networks can be then applied to targeted supervised tasks (e.g., text classification) or to generate new text (Brown et al., 2020). Self-supervision has also resulted into state-of-art speech recognition models by learning directly from speech in multiple languages (Baevski et al., 2020). Together with the reinforcement learning, self-supervised learning is a powerful way to learn underlying rules of the data and construct the intuitive mental models of the environment. In the next section, we describe how the brain works from multidimensional information-processing perspectives.

## Brain as a Prediction Machine

The intuitive mental models allow us to explain why humans are good at solving novel tasks fast with only few examples. However, how does the brain apply and update such models? How does the brain handle the vast richness of the input data? The intuitive models themselves are useless without an efficient method to make valid predictions based on those models. Next, we concentrate on these properties of the brains from new neuroeconomics science perspective. Neuroeconomics highlights how the brain controls human decision making and behavior by using key ideas from psychology, economics, neuroscience, and computational models. In the vein of consilience and multidisciplinary, this approach helps to understand the processes that connect sensation and action by revealing the neurobiological mechanisms by which decisions are made and build predictive models relating to human behavior (Wilson, 1999; Glimcher et al., 2004).

Whereas the classical, behavioral, and neuroscientific research rely on relatively small-scale interpretable models which include only two or three explanatory variables, neuroeconomics emphasizes the prediction models, which allow multiple variables and parameters in these models (Yarkoni and Westfall, 2017; Jolly and Chang, 2019; Hasson et al., 2020). Despite the classical models have discovered keen formal explanations of human behaviors (Von Neumann and Morgenstern, 2007), their disadvantage is that they make it difficult to predict human decision making and behavior in a real-life context outside a laboratory (Hasson et al., 2020). However, the recent methodological advances in neuroscience have demonstrated how the information in the brain is encoded with very high dimensionality with respect to both space and time (Haxby et al., 2014; Jolly and Chang, 2019). Next, we discuss human decision making covering Bayesian hypothesis, default and valuation networks, and high dimensionality perspectives.

### The Bayesian Brain and Meaningful Reasoning

Bayesian model specifies how to update probabilistic beliefs about causal structures of a context in the light of new data. According to Bayesian inference, an individual begins with a set of hypotheses of varying probability (the prior distribution). These hypotheses are based on the person’s beliefs (mental models) about the state of a situation. Then s/he evaluates these hypotheses against the evidence or new information about the context. Then s/he uses Bayes rule and updates the probability of the hypotheses based on the evidence and this yields a new set of probabilities called the posterior distribution (Denison et al., 2013). Through Bayesian reasoning, one can use observed data to update an estimate of the probability that each of several possible structures accurately describes the environment (Gershman and Niv, 2010).

An important function of the brain is to make the observations understandable and meaningful to support an individual’s behavior optimal ways. How this is possible based on a formal Bayesian computation? It is generally assumed that the Bayesian model cannot control the complexity of the reality. Thus, Bayesian inference is not tractable in general, has been claimed (Gershman and Niv, 2010). Here, the person cannot search exhaustively through all the possible hypotheses relating to the state and dynamic of the situation. A number of decision options negatively affects choice as computational performance of a human decreases rapidly with the size of the search space leading to a phenomenon referred as “choice overload” (Murawski and Bossaerts, 2016). However, the applications of Bayesian inference in computer science and statistics approximate these calculations using different kinds of mathematical approximation methods (Denison et al., 2013; Gershman, 2021a). These developed approximation methods help an agent to exploit the complex structure of real-world problems. Approximate inferential methods include procedures that use Monte Carlo sampling, bounding methods, and methods that decompose problems into simpler sets of subproblems (Gershman et al., 2015). In addition, likely one of the most common approximating strategies of a human are Bayesian inference by sampling hypotheses which can also explain why human choices are typically not optimal nor follow actual probabilities. Rich, realistic tasks, in which there is a lot of contextual information available to guide sampling, are just those where the Bayesian sampler is most effective (Sanborn and Chater, 2016). By using this approximation, the sample-based inference converges toward the true posterior as more hypotheses is sampled. The previous studies have found that humans use this strategy across several domains, including category learning, causal reasoning, and perception (Gershman et al., 2015).

It is important to emphasize that the probability judgment in Bayesian sense is not necessarily purely syntactic or computational. Rather, it is sensitive to semantic properties of the combination formed by observation and prior experience, e.g., probability judgments are sensitive to semantic properties of the joint distribution (Gershman, 2021b). The interpretation of the objects and their relationships (e.g., co-occurrence or joint probability) are closer to the Bayesian ideal when the occurrence of probabilities of these objects are believable or meaningful. Hence, it is better to think of probabilities in Bayes’ model as degrees of belief rather than descriptions of randomness (the frequencies of repeating events) (Gershman, 2021a). For example, in the study by Cohen et al. (2017), the participants made judgments about the medical conditions after they got information about the results of a diagnostic test. They found that the people diverged considerably from Bayes’ rule when the probabilities were unbelievable. For example, a medical test with a false positive rate of 80% would be considered unbelievable, because no such test would ever be used in the real world. Similarly, a 50% frequency of occurrence for pneumonia would be considered unbelievable, because it is not the case that every other person you meet has had pneumonia. A similar deviation from the logical reasoning has been observed in syllogistic reasoning, where beliefs about the plausibility of statements influence truth judgments (Revlin et al., 1980). However, arguing that the people revise their beliefs in a way that is consistent with Bayesian inference does not necessarily imply that a human work through the steps of Bayes’ rule in their daily life. It is simply not sensible and useful from either a formal or a practical standpoint to evaluate all possible hypotheses each time when new data are observed.

Furthermore, the semantic properties of judgment are relating to plausible reasoning (Jaynes, 2003). Let us assume that there is a broken window of a jewelry shop and a criminal-looking person is near a broken window. Then a police officer comes to the scene and sees the broken window and the criminal-looking person close to this window. In this situation, she inferences almost immediately that a criminal looking person is the guilty. Jaynes (2003) emphasizes that the police’s decision making is neither deductive nor inductive, but it is plausible reasoning. Despite the plausible reasoning is not necessarily sure, it has a very strong convincing power, and a human decides and inferences this way all the time.

We argue that the plausible reasoning is same as a meaningful reasoning (Suomala, 2020) in which a human uses past experiences, like personal history, cultural habits, and learnings during education, relating a specific context and makes meaningful interpretation about this context by combining the observations with prior experiences according to Bayesian rule. In this way, the model considers the limitations of human mind/brain. The prior mental models help to constrain the most typical and most meaningful decision-making strategies in the different situations (Suomala, 2020). Hence, human behavior is biased to culturally and socially transmitted values. Then, each person anticipates the future situations according to meaningfulness, and this leads to the domain-specific decision-making strategies. For this reason, we argue that the intuitive culture is an essential part of mental models.

Because of the complexity of environments, a human need to represent information efficiently and this often leads to cognitive biases distortions in reasoning and representations (Korteling et al., 2018). Researchers have documented many ways in which individual judgments and decision making depart from rational choice and information processing (Milosavljevic et al., 2012). Despite these cognitive biases contain errors with respect to an objective description of reality, they may be optimal from the subjective perspective of the computational system. From the subjective perspective of human’s mental models, the use of cognitive biases is not an error at all. Rather, it is an indispensable property of complex biological and artificial inferential system (Lieder and Griffiths, 2020; Gershman, 2021a).

As conclusion, meaningfulness is defined as the set of constraint a human’s brain can make with respect to the distinctions between observations (stimuli). Thus, the computational meaningfulness is the results of the subject’s efforts to interpret the properties of context in which s/he behave. Ratneshwar et al. (1987) suggest that the meaning of observation is a function of human’s ability to differentiate stimuli from one another on a given set of observations. To do that, humans need the capacity to concentrate on the most meaningful features of the environment to behave optimal ways in his/her environments (Ratneshwar et al., 1987; Suomala, 2020).

### Brains as Over-Parameterized Modeling Organ and the Role of Default-Mode Network

The main task of the brain is to extract dynamic, multidimensional information about the world to produce rich, context-dependent behaviors, and decisions (Gallistel and Matzel, 2013; Hasson et al., 2020). It is genetically specified information-processing organ for the construction of a contextual probabilistic representation of the world. Each cubic millimeter of human cortex contains roughly 50,000 neurons that may have connections and supports for thousands adjustable synapses with their neighboring and distant cells. This yields a massive set of adjustable parameters; about 300 million in each cubic millimeter of cortex, and over 100 trillion synapses across the entire brain (Hasson et al., 2020). We can assume, that a human brain, based on this huge multidimensional processing of information, as a wildly over-parameterized modeling organ (Conant and Ross Ashby, 1970; Hasson et al., 2020).

Because of the complex and temporally extended nature of observations, incoming stimuli should activate a broad and diverse set of brain regions, especially the brain regions that have encoded the previous experiences. Moulton and Kosslyn (2009) hypothesized that the hippocampus, which is involved in episodic memory retrieval, prefrontal cortices involved in top–down processing and the retrosplenial complex involved in associative processing, are regions which retain prior experiences of a person [see also Hassabis et al. (2007)]. The naturalistic experiments have showed that the default mode network (DMN), involving such regions as medial prefrontal cortex, precuneus, and angular gyrus, play central role in integrating new information with the prior knowledge to form distinct high-level event representations (DuBrow et al., 2017; Kauttonen et al., 2018; Yeshurun et al., 2021). The DMN is considered a major hub for actively processing incoming external information and integrating it with prior knowledge in the social world (Yeshurun et al., 2021).

The traditional way to study human behavior is to focus on two or three artificial explanatory parameters. The basic assumption has been that humans’ have capacity to utilize all available information in the situations created in the experiments. Apparent risk of applying low-dimensional models is that apparent importance of a variable within a model may be inaccurate due to the other unobserved variables. The development of machine learning methods and neuroeconomics have expanded the scope of human behavior research from simple experiments to the more real-life contexts, in which a participant process multidimensional real-like information (Kauttonen et al., 2015; Jolly and Chang, 2019; Hasson et al., 2020). In this way, it is possible to make more accurate predictive models to better match the human behavior, as discussed in the next section. Using computational models will likewise enable both researchers in neuroeconomics and engineers to capture this high dimensionality of human’s decision making to create human-like AI.

The human brain must integrate prior experiences and observations flexibly and efficiently to decide optimal behavior. We argue that the brain’s valuation network is plausible candidate for this work because the activation patterns on this region also predicts an individuals’ and groups of individuals’ behavior outside of the experiments (Genevsky et al., 2017). The neuroimaging studies have demonstrated that the valuation networks are involved in computing relative values of real (e.g., microloans, Genevsky et al., 2017) and abstract things (e.g., moving dots, Magrabi et al., 2021). The brain creates meaning when it uses the valuation network for integrating about aspects of prior experiences and observations. While DMN is the hub for keeping track and integrating ongoing information, the valuation network is specialized in computing values. By finding meaningful decisions and behavior, the brain needs to use approximations and very likely applies Bayesian rule. Evaluating different options in a specific context is costly in time and other resources; thus, the intuitive mental models help a person to concentrate most meaningful options and this way to allocate the scare mental biological resources to decision making (Gershman et al., 2015).

### The Brain’s Valuation Network

Growing evidence from neuroeconomics shows that there are general decision networks in the brain, which count the total valuation of different objects and their relationships using a common neurophysiological “currency” (Levy and Glimcher, 2012; Lim et al., 2013). This serves the same purpose as loss functions applied in training artificial intelligence systems to compare predicted values vs. real values (e.g., cross-entropy and mean-squared error). Whereas several objects and their dynamic interactions with their attributes are involved in these contexts, this complexity makes it almost impossible to isolate and measure the contribution of each object in isolation. However, the brain’s valuation network completes this demanding task and forms a net value of commodities and other items in different contexts from subject’s prior experience perspectives. The activation profile’s changes in this valuation network correlate with an object’s values in a wide class of objects, from simple visual association tasks (Magrabi et al., 2021), biological needs like food (Levy and Glimcher, 2012), clothing (Lim et al., 2013), and money (Glimcher, 2014) to abstract the cultural values like charitable donations (Genevsky et al., 2013) and microloan appeals (Genevsky and Knutson, 2015).

Functional magnetic resonance imaging (FMRI) measures the hemodynamic response related to neural activity, when the participants are lying inside a large camber and see different stimuli during FMRI-experiment. The FMRI measures the blood oxygen level-dependent (BOLD) signal, which varies by different regions in the brain such that blood delivered to an active brain region requires more oxygen than the blood delivered to an inactive region. By using FMRI, it is possible to measure the ratio of an oxygenated to a deoxygenated hemoglobin, when the oxygenated blood produces a stronger magnetic field than non-oxygenated blood (Ashby, 2011). This technology provides researchers the opportunity to study neural activity in the human brain almost real time. Hence, it is no wonder that neuroimaging by FMRI has grown to become the dominant measurement technique in the neuroscience and neuroeconomics (Ruff and Huettel, 2014; Suomala, 2018). It is a non-invasive way of monitoring the mechanisms that underlie how people valuate stimuli, including marketing and health messages, with the potential to shape the thoughts and behaviors of a large population of people (Doré et al., 2020).

Several studies have shown that the responses within regions of the brain associated FMRI-based studies are essential, when the goal is to find forecasting models of human behavior (Genevsky et al., 2017). Consensus of the neuroeconomics research is that the valuation network is formed of the medial prefrontal cortex (MPFC) and ventral striatum (VS). Some of the studies have also connected precuneus to this network, which also serves as the core of the DMN. The MPFC is located in the middle of the frontal lobe and has extensive connections to other areas of the brain. Instead, the striatum is located in the areas below the cortex. The striatum has many connections to the MPFC, and they act together when a human forms total value of some stimuli.

Next, we review the selected seminal, empirical studies in which the activation patterns of data collected from the brain during the FMRI-experiments has demonstrated strong predictive power for human’s behavior also outside of laboratory.

#### Valuation Network Signal as a Robust Predictor of Human Behavior

The properties and anatomy of the valuation network were verified in series of FMRI studies in 2010s. The studies listed in Table 2 demonstrated not only the existence of the valuation network but also how its signal can predict the human behavior in realistic tasks. The predictive power of valuation network signal is often better than the behavioral measurements and self-reports. We argue that the understanding valuation network is valuable in designing human-like AGI. In the following, we briefly summarize these experiments and their key findings. These studies present converging evidence that high-dimensional neural data measured with FMRI carries information that accurately predicts behavior of population.

**TABLE 2:** Selected neuroscience studies that demonstrate the existence of the brains’ valuation network and how its signal can predict real behavior of humans.

Predicted behavior	Key results	References
Sunscreen usage	Neural signals in the MPFC predicted changes in sunscreen use 1 week after scanning. Prediction was 23% more accurate compared to self-reported attitudes and intentions.	Falk et al., 2010
Inclination to quit smoking	Neural signals in the MPFC predicted reduction of smoking 1 month after scanning. Neural prediction was better at population level than self-reports.	Falk et al., 2011, 2012
Online music purchases of adolescents	Activation patterns in brain’s valuation network predicted consuming of previously unknown popular songs and the success of new songs.	Berns and Moore, 2012
Chocolate sales in supermarket	Brain activation patterns in valuation network forecasted better the real supermarket sales of chocolate bars than the participants’ behavioral judgment.	Kühn et al., 2016
Online microloan money lending	Both NAcc and MPFC activities predicted individual lending choices and NAcc activity forecasted loan appeal success on the Internet. The predictive power of neural signals was greater than those of the behavioral choices.	Genevsky and Knutson, 2015; Genevsky et al., 2017
Value estimates of abstract objects	Valuation network incorporates the contextual information and valuation is a dynamic, continuously updated process.	Magrabi et al., 2021

The studies by Falk and colleagues were the first to directly demonstrate link between the valuation network signals and real behavior. In the study by Falk et al. (2010), the participants were exposed to persuasive messages concerning risks of sun exposure. Moreover, neural signals in the MPFC predicted variability in sunscreen usage among participants more accurately than self-report measures like intentions and attitudes measures explained alone. By using a cross-validation, the study revealed that MPFC activation predicted 23% more of the variance in behavior than did self-reported attitudes and intentions to wear sunscreen 1 week following the experiment. Next, Falk et al. (2011, 2012) examined smokers’ neural responses to antismoking advertisement campaigns and subsequent smoking behavior. Consistent with the findings of the sunscreen study, the MPFC activation patterns in the participants’ brain (*n* = 28), when they exposure to anti-smoke message in the scanner, more accurately predicted participants’ inclination to quit smoking 1 month after the initial FMRI than traditional behavioral measurements (Falk et al., 2011). In addition, the activity in the same region of the MPFC that predicted individual smoker’s behavior change during message exposure predicted population-level behavior in response to health messages and provided information that was not conveyed by participants’ (*n* = 31) self-reports (Falk et al., 2012). Neural activity in MPFC predicted the population response, whereas the self-report judgments did not. These results extend the use of FMRI to predict behavior, as opposed to simply predicting immediate effects showing that the critical valuation area in the brain (MPFC) may serve as an indirect marker of future behavior change.

In 2016, Kühn and colleagues did FMRI experiment to test what kind of chocolate commercials promote most sales in the grocery store (Kühn et al., 2016). Researchers showed six versions of a well-known chocolate brand to the participants (*n* = 18) in the FMRI-scanner. After FMRI-scan, the participants in the study were asked behaviorally which advertisement they liked the most. After the FMRI data was acquired chocolate brand were tested at a point-of-sale of the product in a German supermarket; thus, allowing a direct comparison of the sales between the different advertisements tested. Again, the sample’s mean brain activation patterns in valuation network forecasted better the real sales of chocolate bars in supermarket, whereas the participants’ behavioral judgment did not (Kühn et al., 2016). The predictive power of the valuation network was confirmed also for adolescents by Berns and Moore (2012) for music purchases. In this study, the teenage participants (*n* = 28) did listen 60 previously unknown popular music clips in the FMRI-scanner. Songs from 165 relatively unknown artists were used to test the effect of new songs on the participants’ brain, and to test whether the neural signals are predictive of success of songs in the real market. After listening to each song, the participants rated the song based on how familiar it was and how much they liked it; thus, the researchers had both behavioral and neurophysiological data from the participants’ preferences of new songs. The correlation between behavioral subjective song ratings with sales data was near zero (*r* = 0.11). However, the activation within the striatum—one essential region of valuation network in the brain—was significantly correlated to the sales. This research demonstrated that not only the signals in valuation-related networks of the human brain are predictive of one small sample’s purchase decisions but also predictive of population effects.

Genevsky and Knutson (2015) sought to link brain activity in laboratory samples (*n* = 28) to forecasted microloan success on the Internet. Researchers found that while both essential region of the valuation network NAcc (The Nucleus Accumbens; part of the Striatum) and MPFC activities in response to microloan appeals predicted the individual lending choices within a sample, only the sample’s average NAcc activity forecasted loan appeal success on the Internet. Noteworthy, the forecasting power of the sample’s average NAcc activity was greater than the sample’s behavioral choices (i.e., whether they like to invest or not). However, the sample’s ratings of positive arousal in response to the loan appeals continued to forecast loan appeal success on the Internet (Genevsky and Knutson, 2015). In the same vein, crowdfunding study (Genevsky et al., 2017) confirmed the essential results of microloan appeal success study. Findings demonstrate that a subset of the neural predictors in the valuation network of individual choice can generalize to forecast market-level microloan appeal and crowdfunding outcomes—even better than choice itself.

Finally, a recent study by Magrabi et al. (2021) demonstrated that the valuation network incorporates the contextual information and valuation is a dynamic, continuously updated process. Unlike the previous studies, because of the increased complexity of the research question, the stimulus involved abstract dynamical objects (dot clouds) instead of a realistic task. In the FMRI-scanner, participants (*n* = 24) were presented with a dot stimulus varying in the following two constituent perceptual attributes: Motion direction and dot colors. Each attribute level was associated with a specific monetary gain or loss. In the FMRI scanner, the participants had to identify the attribute values and integrate them as the sum of attribute values indicated the overall value of the stimulus and then either accept or reject the monetary offer. The researchers found that the computation of particular attribute values was accomplished in a dynamic manner within the same network comprising posterior cingulate cortex (PCC), posterior inferior temporal gyrus (PIT), and ventral striatum. The results indicate that the attribute values are computed in an interdependent and contextualized manner, such that the attribute values are not computed sequentially and in isolation. Instead, there is a constant exchange of information in which value predictions are continuously updated and re-evaluated.

### From the Activation Patterns of the Valuation Network to the Whole-Brain Patterns

We described above the studies that demonstrated the central role of the brain’s valuation network in human’s decision making and behavior. These studies found that considering information from the valuation network of the brain explains significant variance in out-of-sample message/stimuli effects. However, there also theoretical critiques (Camerer, 2013; Hayden and Niv, 2021) and empirical findings (Doré et al., 2020), which broaden the brain’s valuation networks approach. The deeper analysis of these critiques is out of the scope of this article; however, we review shortly the empirical findings of Doré et al. (2020) from machine learning and engineering perspectives.

Essential assumption of predictive models of human and other complex system behavior is, that the classical empirical explanatory models cannot predicts systems behavior with only a few explanatory variables (Yarkoni and Westfall, 2017; Jolly and Chang, 2019). In the same vein, the current study by Doré et al. (2020) showed that signals from whole-brain patterns—detected by FMRI—associated with reward valuation beyond activity in the valuation network (i.e., striatum and MPFC). Moreover, the study shows that a reward-related pattern of whole-brain activity is related to health message sharing on social media through a population.

Despite the valuation network in the brain has millions of neurons and has properties of an over-parameterized systems, we do not know at this moment, how much brain signals we need to take in the account to build optimal and predictive human behavioral model. Whereas Genevsky et al. (2017) have shown (see above the microloan appeal study) that the signals from the striatum detected by FMRI predicts better the human choice than the signals from whole brain on population level, Doré et al. (2020) showed, on the contrary, that the signals from the whole brain are more predictive of the human behavior on social media at the population level.

As conclusion about FMRI-studies relating to the predictive models, we make two conclusions from engineering (AI research) perspectives. First, it is safe to assume that human brain can operate on many different contexts and multiple timescales. If we like to predict human behavior, we need to understand, how does this complex biological system work and current neuroimaging tools—especially FRMI—gives an opportunity to understand the logic of this complex organ. When this rich, high-dimensional data is analyzed with current machine learning methods and compared against AI model candidates, we are closer in resolving how neural processing (particularly valuation) works. Second, the human brain makes predictions based on its values or its subjective experiences from previous events of meaningful behavioral practices in different environments. Whereas it is difficult to know, what are most meaningful behavioral practice in a current situation, we can detect these values from brain, if we present the most essential real-life stimuli for subjects in the FMRI experiments. There is still an open question, do we need to whole brain or can we concentrate on specific parts, such as valuation and default mode networks, when we try to build better predictive model of human behavior.

## Comparing Brain Model With Current Artificial Intelligence-Models

Information representations in the brain are dynamic mental models, which include causal roles and functions of objects. These mental models help a human understand different scenes in meaningful ways. These mental models cover intuitive physics, intuitive psychology, and intuitive culture. Scene understanding by using dynamic mental models in a specific context is lacking in today’s narrow AI models. Here, we have focused to study especially for the brain’s valuation network, which support individuals to behave and make decisions in optimal ways. However, we also considered the extent to which the brain is involved in decision making in addition to the valuation network (see Doré et al., 2020). Most of the human behavior is a function of a person’s subjective experience of meaningfulness and situational factors, e.g., cultural values and artifacts. Next, we discuss the current state of AI and its limitations and how neuroeconomics and understanding of the brain models could help us overcome these limitations and advance development of AI.

### Bottlenecks and Limitations of Current Artificial Intelligence Models

The first generation of AI models were constructed based on idea that it can utilize all available information by exhaustive enumeration of all relevant properties of the context. The promise of this approach was that it is possible to develop AI that might 1 day both explain and replicate aspects of human intelligence (Gershman et al., 2015). However, these classical models did not consider, that each complex system—biological or artificial—not only uses the resources (exhaustive enumeration) but must allocate the resources in a sensible way (Lieder et al., 2018; Steverson et al., 2019). The current approach for an intelligent behavior emphasizes that the rationality is the efficient allocation of resources. When sampling (search for new information) is costly and an individual believes that most gains or losses are small, as in many everyday tasks; then, rational behavior can be to sample as few as one or a few high-posterior probability hypotheses for each decision from Bayesian perspective (Gershman et al., 2015).

In the face of the complex situation and solving real-world decision making, the new resource-rational approach has emphasized the role of intuitive mental models that might be developed by computer-based reasoning systems to cut through the complexity of decision making. Like in cognitive science, a probabilistic renaissance swept through mainstream AI research, in part by pressures for performing reliable inference about likelihoods of outcomes in applications of machine reasoning to such high-stakes domains as medicine (Gershman et al., 2015). Attempts to mechanize the probability, especially Bayesian inference, for decision and learning led to new insights about probability and stimulated thinking about the role of related strategies in human cognition. For example, the advances in AI led to the formulation of rich network-based representations, such as Bayesian networks, broadly referred to as probabilistic graphical models (PGMs) (Koller and Friedman, 2009). In particular, a belief updating process was developed to efficiently update parameters of the network using parallel and distributed computations (Friston et al., 2021). Belief updating is a process that transforms prior beliefs into posterior beliefs when new information is observed and it is a core mechanism of Bayesian reasoning. Some studies have identified potential neural mechanisms of Bayesian belief updating in human brain at least for spatial attention task (Vossel et al., 2015). It remains to be seen if this also holds for more complex decision’s tasks.

In the recent years, machine learning has been able to solve difficult pattern recognition problems. Such developments have put the notions of backpropagation, using large data sets and probabilistic inference with classical decision-making theory (Von Neumann and Morgenstern, 2007) at the heart of many contemporary AI models. Together with increasing computational power and data set availability have led for AI successes in the recent years. Speech and natural language understanding, self-driving cars, automated assistants, and mastering complex games like Go are some examples of the success of these approaches (Gershman et al., 2015; Schrittwieser et al., 2020; Bengio et al., 2021; He et al., 2021). Although these AI-applications have reached human-level performance on several challenging benchmarks, they are still far from matching human-level behavior in other ways. Deep neural networks typically need much more data than people do to solve the same types of problems, whether it is learning to recognize a new type of object or learning to play a new game. For example, while humans can learn to drive with few dozen hours of practice, self-driving cars need millions of (simulated or real) hours and still lack behind human performance in handling surprising situations (Lake et al., 2017). Or when learning the meanings of words in their native language, children easily make meaningful generalizations from very sparse data. In contrast, AI based deep reinforcement learning systems still have not come close to learning to play new games like Atari as quickly as humans can (Lake et al., 2017).

The main challenge in AI-development is to move from the classical view of a rational agent who maximizes the expected utility over an exhaustively enumerable state–action space to a model of the decisions faced by resource–rational AI systems deployed in the real world, which place severe demands on real-time computation over complex probabilistic models (Gershman et al., 2015). The intuitive models, as described in “Intuitive Mental Models,” allow humans concentrate to most meaningful aspects and behave optimal ways in different contexts. Although great steps have been made in the development of AI, people are still learning from fewer data—often to see just one or a few examples—and form dynamic mental models in richer and more flexible ways than AI (Bengio et al., 2021).

The current AI systems have bottlenecks when working in real-world setting. First, they are prone to outliers and can make trivial mistakes (from a human perspective), such as self-driving car confusing regular stop-signs posts and those printed on billboards^1^ or being held by a human,^2^ image-classifier getting fooled by written texts^3^ and failing to recognize partially occluded objects (Hendrycks et al., 2021). Adding a structured noise invisible to humans into images can lead to a complete failure in the state-of-the-art image recognition models (Ren et al., 2020). Second, AI models can be biased, resulting in underpowered predictions with possible toxic outcomes (Seyyed-Kalantari et al., 2021; Weidinger et al., 2021). Such flaws can be considered as symptoms of lacking intuitive, commonsense world models that would allow AI to have more complete understanding of the world and generalize over novel situations (Bengio et al., 2021).

### Brain Models and Importance of the Valuation Network

As we have described in this article, human brain is evolved to grasp and learns basic understanding of systems of abstract concepts—represented as intuitive theories in the brain for physics, psychology, and culture. Thus, a human brain can understand easily the physical objects and substances, intentional agents, and their causal interactions in time and space (Lake et al., 2017). The current computational rational model of brain proposes that humans decide and behave by using principles of Bayesian model in the uncertain and ambiguity contexts (Gershman et al., 2015; Suomala, 2020; Friston et al., 2021). Furthermore, the current approach in neuroeconomics is that the human brain’s goal might be to learn about the structure and functions of environment rather than simply maximize a reward (Denison et al., 2013). This learning approach is consistent with the idea that brain computes values for environmental objects, particularly if we consider meaningfulness and new information as essential dimensions of valuation in addition to maximizing expected utility in traditional economic sense (Camerer, 2013; Gershman et al., 2015). This learning approach is not an alternative for traditional models, rather it may be viewed as an extension of them.

We believe that the future generations of AI will look different from the current state-of-the-art neural networks, because it may be endowed with an intuitive physics, an intuitive psychology, and an intuitive culture (Lake et al., 2017). Studies have shown that the small samples of participants’ brain activation profiles in valuation network can predict real behavioral chance in a real context outside of laboratory on individual levels (Berkman and Falk, 2013) and on population level (Falk et al., 2012; Genevsky et al., 2017). We argue that in reverse engineering, the mechanisms of brain’s valuation and default mode networks will inform the development of human-like AGI. These neural systems hold solutions of dealing with high-dimensional input, keeping track and integrating ongoing contextual information and computing relative values to make informed decisions. Computations of the valuation network in particular could be the solution of advancing AI. For example, is there a biological equivalent for gradient-based backpropagation algorithm that uses valuation and prediction errors to guide behavior and learning? In Section “Brain as a Prediction Machine,” we described how studies have shown that this brain network has critical role, when a human decides and behaves. We need further research to better describe the mechanisms of the brain’s valuation network in an engineering way.

Despite the recent achievements of AI, people are better than machines in solving a wide range of difficult computational problems in their real-life contexts and behaving in (subjectively) optimal ways by taking advantage of information in inherently complex, uncertain, and continuously streaming inputs. Capturing more human-like, flexible behavior, AGI systems might first need to adopt the brain’s capability to form dynamic mental models with the intuitive models and the valuation-like networks described above.

## Discussion

We think it is very unlikely that a revolutionary artificial intelligence will emerge through engineering and computer science alone. Observational findings coming from neuroscience and behavioral sciences are also needed to develop new algorithms that can lead us closer to human-level artificial general intelligence.

Although great steps have been made in the development of AI, for example, in machine vision (e.g., self-driving cars), text and speech understanding (e.g., virtual assistants), and playing games (e.g., chess, poker and Go), AI is still far from human ability to learn as efficiently and master multiple different tasks (Bengio et al., 2021; Gershman, 2021a). People are still learning from fewer data—often to see just one or a few examples—and form dynamic mental models in richer and more flexible ways than AI. We believe that the future generations of AI may be endowed with intuitive physics, intuitive psychology, and intuitive culture (Tomasello et al., 2005; Lake et al., 2017). While these do not necessarily cover all intuitive models that humans can possess, these are sufficient starting point in development of a human-like AGI. The current AI models can be prone to trivial mistakes and biases. This is a symptom of current AI models missing contextual and commonsense understanding of the world in both local and broad senses. For example, for a self-driving car to adapt from operating in sunny California to winterly Scandinavia needs to grasp both different physical conditions and rules (laws) of the environment. Therefore, we argue that the intuitive culture that covers large-scale nuances of the environment is very important.

We can pinpoint the following three major gaps between the current AI models and human optimal behavior. First, AI is effective in solving recognition problems but incompetent in building causal models of the world that support explanation, understanding, and prediction. Especially, the scene understanding and the relationships of objects’ functions in a specific context is lacking in today’s AI. For example, when image captions are generated by a deep neural network, it gets the key objects in a scene correct, but fails to understand the relationships between objects and people in the image, or the causal relationships between the objects (Gershman, 2021a). However, a human can apply dynamic mental models. Second, AI cannot generalize its knowledge to new tasks and situations. When an infant can learn and predict the movements of wooden blocks based on few examples, AI (PhysNet) requires extensive training—between 100,000 and 200,000 scenes—to answer the question, “Will the tower fall or not?” (Lake et al., 2017). Thus, a human can generalize to many novel complex scenes with only few training samples or trials. Third, a current AI neglects the contextual information in its applications whereas a human’s brain constantly benefits the information in a specific context. This poses a big problem since context carries often critical information that can greatly affect the decision. Training a new AI model for each context is not feasible, instead the context needs to be inherently build-in within the model.

Computational meaningfulness offers a potential unifying framework for the study of optimal behavior of artificial agents. The parts of this framework that we consider essential for development of such agents are listed in Table 1. We argue that valuation and default mode networks which have gained lots of interest in neuroscience research past decade offer a novel viewpoint for the development of AI. We argue that studying and reverse-engineering the related neural computations are needed in the development of new algorithms for both intuitive model learning and usage. With this work, we aim to bring this research to the attention of engineers working on AI and make connections between the fields.

The human brain as resource rational agents that seek to form dynamic mental models by effectively apply Bayes’ rule and approximate algorithms that support meaningful actions in each situation (Cohen et al., 2017; Gershman, 2021b). This process incorporates the costs of computation and consider in optimal way the human’s specific biological, social, and cultural needs. When the science uncovers this process and its elements better than today, we can apply this process at least partially through engineering design to build better human-like machines, including AGI. Thus, the science of human decision making, e.g., neuroeconomics is the foundation of next generation of AGI. Neuroeconomics often considers decision making in real-life scenarios, which incorporates also cultural aspects. We should concentrate on how such decisions are computed by the brain. Essential question in future is how the brain’s computation can be captured in engineering terms. We have described studies (see Section “Brain as a Prediction Machine” and Table 2) in which these ideas are being fruitfully applied across the disciplines of human behavior, but we admit that a genuine unifying theory about human decision making and its application to AGI remains mostly a promise for the future. Especially, the question about the mechanisms of the brain for cost-sensitive meaningful computation in valuation networks and it’s applied to AGI will be essential in future (Gershman et al., 2015).

The better we understand human’s brain mechanisms, the better we can apply this understanding for building algorithms and models that gets us closer to human-like AGI. On the other hand, the science also benefits the development of AGI by applying theoretical and methodological ideas from algorithms development and big data analysis. According to Glaser et al. (2019) AI can help neuroscience at least in the following ways: Solving engineering problems (e.g., building better predictive models), identifying predictive variables (e.g., apply regularization and find causal relationship), benchmarking simple models (e.g., linear vs. non-linear), and serving as a model of the brain to compare against algorithms. Due to the complexity of large datasets that can be both non-linear and recurrent, it is necessary to apply machine learning methods that can extract meaningful relationships and structure (Glaser et al., 2019). It has become evident that the classical statistical modeling, such as general linear regression, that rely on inference rather than predictive power, is insufficient when trying to find working principles of brain (see, e.g., Jolly and Chang, 2019).

Finally, we acknowledge that the limitation that the computational meaningfulness framework we described here is still a concept and not an algorithm or computational model that can be directly applied to create a next-generation AI models. Instead, we hope that intuitive models, contextual Bayesian inference, and valuation and default mode networks will enhance dialogue between research fields and inspire development of new generation of computational algorithms in engineering.

## Summary and Conclusion

In this study, we have discussed the key concepts for development of human-like artificial general intelligence (AGI). These concepts include learning intuitive mental models *via* reinforcement and self-supervised learning, and using and updating these models *via* Bayesian inference. We also discussed about the default mode and valuation networks of the human brain engaged in keeping track of ongoing events, contexts, and evaluating the relative values of things and decisions. We have pinpointed major shortcomings of the currently available AI models related to lacking intuitive mental models, inflexible generalization between tasks and lack of contextual information relevant for optimal decisions. We argue that the intuitive culture is a necessary element of a human-like AGI as it defines the contexts for optimal decisions of real-life actions. We also argue that reverse-engineering the core working principles of default mode and valuation networks is the key to unlocking mechanism behind contextual, high-dimensional input signals processing, and computation of value (reward) signals. This requires a close interplay between engineering, computer science, neurosciences, and behavioral sciences with collection of big observational datasets, e.g., *via* FMRI. Making distinction between observations in the environment and concentrating on the most meaningful features are essential for the optimal behavior in the environment. We call this framework, which applies intuitive models and Bayesian inference principles to make contextual decisions, as computational meaningfulness. We hypothesize that the computational meaningfulness allows machines to reason and make decisions like those by humans and it is a promising path to human-like AGI.

## Data Availability Statement

The original contributions presented in the study are included in the article/supplementary material, further inquiries can be directed to the corresponding author.

## Author Contributions

Both authors wrote the manuscript, contributed to the article, and approved the submitted version.

## Conflict of Interest

The authors declare that the research was conducted in the absence of any commercial or financial relationships that could be construed as a potential conflict of interest.

## Publisher’s Note

All claims expressed in this article are solely those of the authors and do not necessarily represent those of their affiliated organizations, or those of the publisher, the editors and the reviewers. Any product that may be evaluated in this article, or claim that may be made by its manufacturer, is not guaranteed or endorsed by the publisher.

## Footnotes

1
https://futurism.com/the-byte/tesla-slamming-brakes-sees-stop-sign-billboard

2
https://nautil.us/deep-learning-is-hitting-a-wall-14467

3
https://distill.pub/2021/multimodal-neurons
